# A case report of mantle cell lymphoma presenting as intussuscepting colon mass

**DOI:** 10.1016/j.ijscr.2020.03.022

**Published:** 2020-03-28

**Authors:** Brandon M. Smith, Kyle Reilly, Elena Baker, Amy Deeken, Adrian G. Dan

**Affiliations:** a Department of Surgery, Akron City Hospital Summa Health, Akron, OH, USA; b Northeast Ohio Medical University, Rootstown, OH, USA

**Keywords:** Case report, Mantle cell lymphoma, Primary GI lymphoma, Colo-colonic intussusception

## Abstract

**Introduction:**

Mantle Cell Lymphoma (MCL) is a non-Hodgkin lymphoma accounting for 2.5% of lymphoid neoplasms in the United States. Primary gastrointestinal (GI) lymphomas account for 1–4% of all GI malignancies, with few reports of primary mantle cell lymphoma presenting as a single colonic mass and none to our knowledge with colon-colonic intussusception as the presenting finding. Accurate and timely diagnosis is imperative because MCL has rapid progression and early chemotherapeutic intervention results in improved patient outcomes. This work is reported in line with the SCARE criteria [1] for case report publication.

**Presentation of case:**

A 61-year-old male presented with 1 month history of nonspecific right sided abdominal pain. Computed Tomography (CT) of the abdomen identified an intussuscepting mass in the proximal ascending colon and an additional 8 mm hepatic lesion. Colonoscopy identified a large mass in the corresponding area of colon identified on CT. Histology and immunohistochemistry of biopsied specimen diagnosed MCL.

**Discussion:**

Planned surgical intervention was deferred and the patient was referred for oncologic treatment. We report the first case to our knowledge of MCL presenting as colon-colonic intussusception and discuss the work-up of this rare lymphoma that clinicians may be required to diagnose and manage.

**Conclusion:**

This report serves as a reminder to maintain a broad differential inclusive of uncommon diseases and unanticipated pathology. Practicing with a thorough understanding of medical principles and clinical acumen is essential for optimal patient care and, as demonstrated in this case, preventing a potentially unnecessary surgical intervention thus delaying appropriate chemotherapy.

## Highlights

• Primary gastrointestinal lymphomas rarely present as a single colonic mass.
• This is a case report of a single mass colonic mantle cell lymphoma causing a colon-colonic intussusception.
• Timely diagnosis and treatment of mantle cell lymphoma is critical.
• Case represents a patient whose treatment plan was unexpectedly altered from surgery after a biopsy.

## 1 Introduction

Mantle Cell Lymphoma (MCL) is a non-Hodgkin lymphoma (NHL) characterized by clonal expansion of malignant B type lymphocytes originating from the mantle zone of lymphoid follicles. Excessive lymphocyte proliferation is driven by over expression of protein Cyclin D1 due to a chromosomal translocation of t(11;14)(q13:q32) resulting in the fusion of CCND and Immunoglobulin heavy chain genes. MCL most commonly presents in the adult population, with initial symptoms including lymphadenopathy, fevers, night sweats, fatigue, and weight loss. MCL represents approximately 2.5% of lymphoid neoplasms in the United States [2] with the most frequent secondarily involved extra-nodal site being the gastrointestinal tract, representing 5–20% of MCL cases [3]. Primary extra-nodal disease is uncommon as MCL only accounts for 1–4% of primary GI tract lymphomas [4]. The most common endoscopic finding in gastrointestinal MCL is Multiple Lymphomatous Polyposis with primary solitary MCL mass lesion of the colon is extremely unusual. Enteric intussusception in the adult population is also relatively infrequent with a majority of cases associated with pathologic lead point. Intussusception caused by malignant lymphoma is unusual, accounting for less than 10% of all cases of colonic intussusception [5].

Herein, we present a case of primary mantle cell lymphoma presenting as a single colonic mass causing colon-conic intussusception, which to our knowledge has never been described in the literature, as well as review the staging and current treatment guidelines of primary gastrointestinal MCL. This work is reported in line with the SCARE criteria for case report publication [1].

## 2 Case presentation

**Fig. 1:**
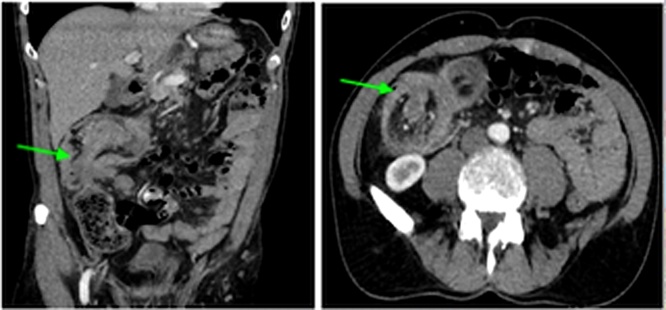
Initial contrast-enhanced computed tomography scans of the abdomen demonstrating colon-colonic intussusception of the cecum and ascending colon.

A 61-year-old otherwise healthy Caucasian male presented to the emergency department for evaluation of 1 month history of generalized intermittent abdominal pain with occasional dark blood with bowel movements. He denied fever, chills, weight loss, diarrhea, or obstructive symptoms. He had no family history of gastrointestinal, hematologic, or malignant diseases. He underwent screening colonoscopy 12 years prior which was unremarkable. Physical examination yielded no significant findings. Complete blood count and basic metabolic panel were within normal limits. An abdominal and pelvic computed tomography (CT) scan demonstrated a proximal colon-colonic intussusception with appearance of a mass at the leading edge of the intussusceptum with diffuse mural thickening in the intussusceptum and the intussuscipiens with extensive mesenteric lymphadenopathy (Fig. 1). Findings were discussed with the patient who refused hospital admission. He was discharged with outpatient appointment arranged.

**Fig. 2:**
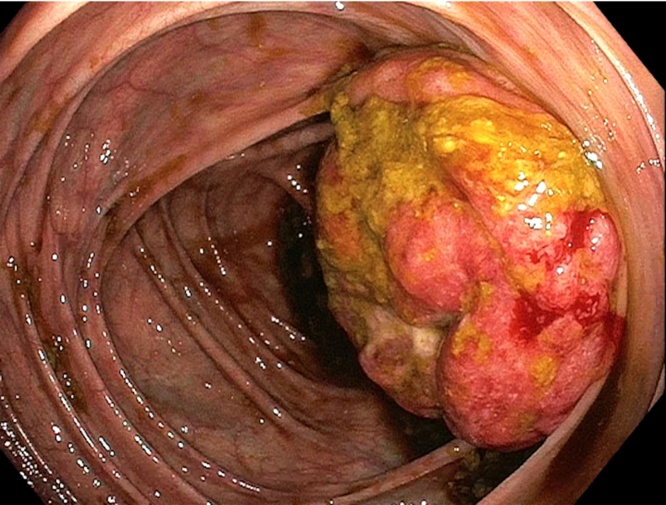
Endoscopic findings: colonoscopy revealing large tumor-like protruding mass near the cecum.

In office 3 days later, the patient reported continued intermittent sharp abdominal pain and denied obstructive symptoms. With a presumed diagnosis of colonic malignancy, work-up was pursued in accordance with National Comprehensive Cancer Network (NCCN) guidelines. Carcinoembryonic Antigen level was 0.5 ng/mL. Staging CT chest demonstrated a 12 mm hyper-enhancing focus in liver Segment 8. Colonoscopy identified a traversable, fungating, non-circumferential 10 cm cecal mass and a 15 mm pedunculated descending colon polyp (Fig. 2). Cold forceps biopsies were obtained and polypectomy was performed. The patient was scheduled for laparoscopic right hemicolectomy and hepatic wedge resection.

**Fig. 3:**
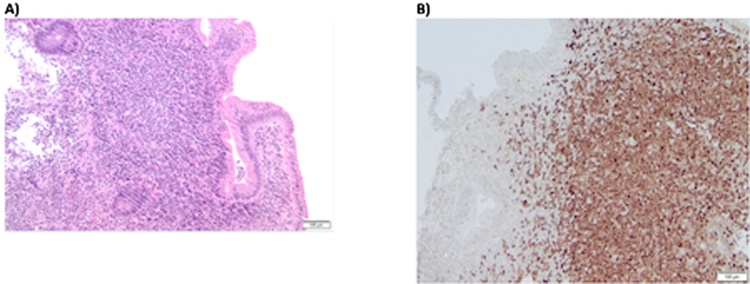
Pathological images from biopsy specimen. **A.** H&E staining of biopsy specimen. (×100). **B.** Cyclin D1 expression in majority of nuclei. (×100).

Biopsy histology demonstrated marked expansion of colonic lamina propria by a lymphoid infiltrate composed predominately of small, mature appearing lymphocytes. Immunohistochemical (IHC) staining identified the abnormal lymphocytes to be CD20+, CD5+, BCL2+, Cyclin D1+, and SOX11+ B-cells with proliferation index of 30–40% by Ki-67+ nuclei (Fig. 3) diagnosing MCL. The abnormal cell population was negative for BCL6 and CD23. The pedunculated polyp was a benign inflammatory polyp.

This case was presented at hospital tumor board which recommended cancellation of surgery and oncologic referral.

## 3 Discussion

Mantle cell lymphoma is a non-Hodgkin lymphoma representing 2.5% of lymphoid neoplasms in the United States and 7–9% of lymphoid neoplasms in Europe [2]. MCL most commonly presents in the adult population, median age of 60 years, with male-to-female ratio of >2:1. Most patients are not cured with current chemotherapy and have median survival of 3–5 years [6]. High Ki-67 proliferation index is associated with adverse prognosis [6].

MCL pathophysiology is well established, characterized by a chromosomal translocation (11;14)(q13;q32), present in >95% of cases. This translocation between immunoglobulin heavy chain (IgH) gene and the CCND1 gene yields an unregulated overexpression of protein Cyclin D1 driving cell proliferation. Cyclin D1 detection serves as a hallmark for histopathologic diagnosis, with cells also demonstrating intense surface IgM/IgD, which is more frequently lambda restricted [6]. Cells have uniform positivity of BCL-2, with CD5, FMC7, and CD43 usually positive. MCL cells are occasionally positive for IRF4/MUM1 and negative for BCL6 and CD10. Additionally, SOX11 is positive in >90% of the MCL cases, including cyclin D1-negative and blastoid variants. Beyond t(11;14), MCL has multiple other less common potential chromosomal aberrations with the t(8;14)(q24;q32) and MYC translocations being associated with adverse prognosis [6].

The Ann Arbor Staging Classification is used for MCL staging [7]. The classification scheme ranges from Stage 1 through Stage 4, corresponding to progressive spread, with additional adjunct designations of involvement that may contribute to any stratified stage. Stage 1 disease involves a single nodal region or a single extra-lymphatic organ with the absence of lymph node involvement. Stage 2 disease involves two or more lymph node regions on the same side of the diaphragm, or localized involvement of a single extra-lymphatic organ with regional lymph node envelopment. Stage 3 disease includes lymph node region involvement on both sides of the diaphragm with or without accompanied extra-lymphatic extension. Stage 4 disease is characterized by diffuse or disseminated involvement of one or more extra-lymphatic organs with or without associated lymph node involvement. Additional adjunct designations including the presence or absence of B type symptoms, contiguous nodal involvement, and splenic involvement are used to further stratify the stage [7].

The National Comprehensive Cancer Network (NCCN) guidelines [8] delineate stage based chemotherapeutic treatment strategies for MCL. Stage 1 and stage 2 disease treatment involves induction therapy with several chemotherapeutic options, most commonly RDHA – rituximab, dexamethasone, cytarabine, and platinum-based agents such as carboplatin, cisplatin, or oxaloplatin. Alternative therapy includes rituximab, cyclophosphamide, doxorubicin, and prednisone (R–CHOP). Treatment of bulky stage 2, stage 3 and stage 4 disease includes RDHA, clinical trials, or high dose consolidation with autologous stem cell rescue, and maintenance rituximab [8].

MCL prognosis is generally poor despite aggressive therapy with median overall survival of 3–5 years [9]. Nearly 80% of patients demonstrate extra-nodal involvement at initial presentation with documented sites including bone marrow, spleen, Waldeyer’s ring, and the gastrointestinal tract [10]. The incidence of secondary gastrointestinal tract involvement ranges from 10 to 28% [11]. Primary gastrointestinal tract involvement is much less common, accounting for 1–4% of primary gastrointestinal lymphomas [4]. The most common form of gastrointestinal involvement in MCL is multiple lymphomatous polyposis (MLP). This is identified at endoscopy with findings of numerous, small, round polyps involving long segments of small or large bowel, most commonly the ileocecal region and more infrequently the esophagus and anus [12,13]. Contrary to the more commonly presenting MLP, our patient presented with a single, large, protruding mass-lesion of the cecum causing intermittent non-obstructing intussusception without systemic or B-type symptoms.

Intussusception is typically seen in children resulting from lymphoid hyperplasia of the terminal ileum. Intussusception in adults is rare with an overall annual incidence of 2–3 cases per 1,000,000 in the general population [4]. When diagnosed, intussusception in adults is almost invariably secondary to a mass lesion serving as a lead point. In the small bowel, the most common mass lead points are benign neoplasms such as lipomas and hamartomas, with 30% of intussusception cases induced by malignancy [5]. In the large intestine, 60% of intussusception cases are induced by malignancy [5].

To our knowledge, this is the first reported case of primary gastrointestinal MCL presenting as colon-colonic intussusception. *Daniel* et al. described a case of a single mass colonic primary mantle cell lymphoma in 2016; however, their patient did not have intussusception [14]. *Matsueda* et al. described a single patient with primary MCL as a single protruding lesion of the small intestine causing ileocecal intussusception as the presenting symptom [15]. *Grin* et al. described two additional cases of ileocolic intussusception attributed to MCL mass lesions, however their reported patients were diagnosed with MCL prior to experiencing intussusception [16].

Our case is unique in that the initial complaint of abdominal pain was caused by colon-colonic intussusception, which was the initial presenting sign of his underlying MCL.

## 4 Conclusion

This case of intussusception attributable to primary colonic MCL as a single mass lesion adds to the literature describing another variable clinical presentation of primary gastrointestinal MCL. Although quite uncommon, primary colonic MCL ought to be included in the differential diagnosis of a single mass lesion in the colon. As demonstrated in this case, adequate tissue sampling of colonic masses is paramount to clarify the diagnosis and direct the treatment strategy. This case serves as a reminder to maintain a broad differential inclusive of uncommon diseases and atypical pathology. Maintaining and practicing a thorough understanding of medical principles through work-up, diagnosis, and treatment is essential for optimal patient care and, as demonstrated in this case, preventing an unnecessary surgical operation, which may delay the appropriate chemotherapeutic intervention.

## Declaration of Competing Interest

The authors state that they have no conflict of interest for this report, declarations of interest: none.

## Sources of funding

There was no study sponsor and no funding provided for this project.

## Ethical approval

This case report is exempt from ethical approval at our institution.

## Consent

Written informed consent was obtained from the patient for publication of this case report and accompanying images. A copy of the written consent is available for review by the Editor-in-Chief of this journal on request.

## Author contribution

Brandon Smith: Conceptualization, Methodology, Writing- Original draft preparation, reviewing and editing.

Kyle Reilly: Conceptualization, Writing- Original draft preparation.

Elena Baker: Writing- Original draft preparation, reviewing.

Amy Deeken: Reviewing.

Adrian Dan: Supervision, Writing- revising and editing.

## Registration of research studies

This is not human subjects research.

## Guarantor

Adrian G. Dan, MD.

## Provenance and peer review

Not commissioned, externally peer-reviewed.
